# Lung life HRQoL measure: psychometric properties and initial data in presumptive TB

**DOI:** 10.5588/ijtldopen.24.0580

**Published:** 2025-03-12

**Authors:** M.G. Anthony, G. Hoddinott, C. Purdy, V. Luke, M. Van Niekerk, A.C. Hesseling, M.M. van der Zalm

**Affiliations:** ^1^ Desmond Tutu TB Centre, Department of Paediatrics and Child Health, Faculty of Medicine and Health Sciences, Stellenbosch University, Cape Town, South Africa;; ^2^ School of Public Health, Faculty of Medicine and Health, The University of Sydney, Sydney, Australia.

**Keywords:** presumptive tuberculosis, health-related quality of life, paediatric TB, respiratory illnesses, psychometric properties

## Abstract

**INTRODUCTION:**

Health-related quality of life (HRQoL) data in young children with respiratory illnesses, including TB, are limited in low- and middle-income countries (LMICs). This study assessed the psychometric properties of the LuLi-Q measures in South African children with presumptive TB, focusing on children aged 0–5 years.

**METHODS:**

In a cross-sectional study within the UMOYA TB diagnostic study, HRQoL data were collected using the LuLi-Q-Tots (0–2 years) and LuLi-Q-Pres (3–5 years) measures. Analyses included descriptive statistics, item–total correlations, and Cronbach’s alpha for reliability.

**RESULTS:**

Among 160 children aged 0–5 years (50 aged 0–2 years, 110 aged 3–5 years), the LuLi-Q-Tots had minimal floor and ceiling effects (6.5%), effectively capturing HRQoL. The LuLi-Q-Pres showed substantial floor and ceiling effects (61%), but removing 29 items improved reliability (Cronbach’s alpha: 0.96–0.97). Caregivers reported daily medication use (54%) and anxiety (72%) in the 0–2 group, while separation anxiety (65%) and jealousy (92%) were common in the 3–5 group.

**CONCLUSION:**

This study establishes a foundation for reliable HRQoL measures for young children with presumptive TB, guiding future research and patient-centred care in LMICs.

Respiratory illnesses have a profound impact on child health, accounting for approximately 9 million deaths annually among children aged 0–17 years globally. The highest mortality rates occur in children aged <5 years, with 90% of these deaths occurring in low- and middle-income countries (LMICs).^1–3^ Pulmonary TB (PTB) often presents symptoms that mirror other respiratory illnesses in young children, making diagnosis and treatment challenging, especially in low-resource settings where microbiological confirmation is not always feasible.^4,5^ While growing evidence highlights the impact of TB on children’s health-related quality of life (HRQoL), most available data focuses on adults.^6–9^

Health-related quality of life (HRQoL) encompasses physical, emotional, social, and psychological health, making it a critical metric for evaluating patient-centered care.^10,11^ Disease-specific HRQoL measures are preferred for their sensitivity to illness-specific changes.^11^ However, HRQoL data on young children with presumptive PTB in LMICs remains scarce. Existing measures are often generic and lack the design considerations necessary for LMIC contexts. Previous research has shown that caregivers’ perceptions of HRQoL in children aged <5 years are heavily influenced by socio-economic factors.^9^

Given the high burden of childhood respiratory illnesses in LMICs, there is a clear need for a reliable, valid HRQoL measure for young children.^12^ This manuscript aimed to conduct a pilot study to perform an initial analysis of the Lung Life Health-Related Quality of Life (LuLi-Q) measures for children aged 0–5 years with respiratory illnesses. Additionally, we conducted a preliminary exploration of HRQoL using these measures in a cohort of South African children with presumptive PTB.

## METHODS

### Study design

This study used a cross-sectional study design, and data was collected within a prospective longitudinal TB diagnostic cohort study called Umoya, as previously described.^13^

### Research setting

Children were enrolled in the Umoya study at Tygerberg Hospital (TBH) and Karl Bremer Hospital (KBH), secondary and tertiary academic hospitals in the Cape Metropolitan District (CMD), Western Province, South Africa. The South African healthcare system faces challenges due to the ongoing high disease burden, social inequalities, and infrastructural problems.^14,15^ Many communities in the CMD face socio-economic challenges, such as unemployment, poverty, and substance abuse.^16,17^ Children are particularly exposed to indoor and outdoor pollution, poor nutrition, incomplete vaccination, and HIV exposure, increasing the risk of respiratory illnesses.^18,19^

### Data collection procedures

Participants were recruited using a database developed during the earlier phase of the study. This MS Excel (Microsoft, Seattle, WA, USA) database included information such as study ID, date of birth, age, and illness category (children with TB and children without TB). It was used to identify all children aged 0–5 years within the larger Umoya study, who were then contacted to participate in the nested study. Once the version 1 item banks were finalised, eligible participants were contacted and invited to participate. Participants were required to complete the LuLi-Q measures, which consisted of two separate questionnaires: one for children 0–2 years and another for children 3–5 years. Both measures focused on assessing the child’s HRQoL. Participants were allowed to complete the questionnaires in person during their routine clinical visit or via telephone, with an average completion time of approximately 20–25 min.

The questionnaires were administered by trained Umoya research counsellors using an interviewer-led approach. During this phase, all questionnaires were conducted in English, a language in which all participants were verbally fluent. Data were captured electronically using case report forms and securely entered into the REDCap database (Research Electronic Data Capture) (Vanderbilt University, Nashville, TN, USA).^20,21^

### Instruments

#### Lung life infant and toddler HRQoL measure (LuLi-Q-Tots)

The newly developed LuLi-Q-tots measure for children between 0–2 years included 107 items across seven domains: physical health (*n* = 31), emotional health (*n* = 7), behavioural expressions (*n* = 18), social well-being (*n* = 12), feeling love (*n* = 7), early development (n = 21), and routine (*n* = 10). The response format was a 5-point Likert scale: 1 = strongly agree, 2 = agree, 3 = neutral, 4 = disagree, and 5 = strongly disagree. The prescribed minimum variability is 1, and the maximum is 5.

#### Lung life preschoolers HRQoL measure (LuLi-Q-Pres)

The newly developed LuLi-Q-Pres item bank for children between 3–5 years included 131 items across seven domains: physical health (*n* = 32), emotional health (*n* = 12), psychological (*n* = 31), social well-being (*n* = 7), feeling love (*n* = 7), preschool readiness (*n* = 21), and routine (*n* = 10). The response format was a 5-point Likert scale: 1 = strongly agree, 2 = agree, 3 = neutral, 4 = disagree, and 5 = strongly disagree. The prescribed minimum variability is 1, and the maximum is 5.

### Data analysis

Questionnaire data were exported from the study database into Excel, and all queries were resolved before further analysis in IBM SPSS Statistics software (version 29.0.2.0). Descriptive statistics were calculated to summarise participant demographics and HRQoL. Participants responded to HRQoL statements using a 5-point Likert scale: ‘Strongly Agree,’ ‘Agree,’ ‘Neutral,’ ‘Disagree,’ or ‘Strongly Disagree.’ Responses ‘Strongly Agree’ and ‘Agree’ were considered as agreement, while the remaining responses were categorised as disagreement with statements.

Floor or ceiling effects were defined as occurring when over 15% of participants achieved the minimum or maximum possible score, respectively.^22^ In this study, item correlations were assessed at the individual level using item analysis. Inter-item correlations (IIC) were classified as follows: < 0.5 (poor), 0.5–0.74 (moderate), 0.75–0.89 (good), and > 0.90 (excellent).^23–25^ Internal consistency reliability was evaluated using Cronbach’s alpha (α).^23–25^

### Ethics considerations

The Umoya study and its sub-components, including the nested quantitative HRQoL assessments, were approved by the Health Research Ethics Committee at Stellenbosch University, Tygerberg, South Africa (N17/08/083 & S21/05/084). Caregiver participants provided written informed consent at recruitment.

## RESULTS

### Characteristics of the sample

A total of 160 questionnaires were completed by participants, comprising (0–2 years: *n* = 50; 3–5 years: *n* = 110) (Table 1). Of the 160 participants, 82 (51.3%) initiated TB treatment based on clinical diagnosis or microbiological confirmation of TB. Among the remaining 78 (48.8%) participants who did not require TB treatment, careful evaluation and follow-up classified them as ‘symptomatic controls.’ The HRQoL questionnaire was administered during routine clinical visits.

**Table 1.** Demographic characteristics of the participants by age group.

	All participants	Cohort 1: Age 0–2 years	Cohort 2: Age 3–5 years
	(*n* = 160)	(*n* = 50)	(*n* = 110)
	*n* (%)	*n* (%)	*n* (%)
Sex			
Male	70 (43.8)	21 (42.0)	49 (45.5)
Female	90 (56.3)	29 (58.0)	61 (55.5)
Age, years, median [IQR]	3.4 [2.4–4.7]	1.9 [1.4–2.4]	4.3 [3.3–5.1]
Ethnicity			
Coloured	99 (61.9)	32 (64.0)	67 (60.9)
Black	60 (37.5)	18 (39.0)	42 (38.2)
Indian	1 (0.6)	0 (0.0)	1 (0.9)
Classification			
Children with TB	82 (51.3)	27 (54.0)	55 (50.0)
Symptomatic children	78 (48. 8)	23 (46.0)	55 (50.0)
HIV status			
Negative	146 (91.3)	47 (94.0)	99 (90.0)
Positive	12 (7.5)	1 (2.0)	11 (10.0)
Unknown	2 (1.2)	1 (2.0)	0 (0.0)

IQR = interquartile range.

The median age of children using the LuLi-Q-Tots was 1.9 years (IQR 1.4–2.4) and 4.3 years (IQR 3.3–5.1) for those using the LuLi-Q-Pres, with a similar proportion of males and females. In total, 12 (7.5%) children were living with HIV (CLHIV).

### Psychometric properties of the LuLi-Q-Tots measure

#### Floor and ceiling effects for the LuLi-Q-Tots measure

A total of (*n* = 107) items across the seven HRQoL domains were screened for floor and ceiling effects. Six items (6.5%) demonstrated floor and ceiling effects of ≥15%. Floor effects, characterised by ‘Strongly disagree’ responses, were noted in two domains: physical health and feeling loved. Among the 32 physical health items, 2 (6.0%) exhibited floor effects, while 4 (57.1%) of the seven items feeling love items showed floor effects.

#### Item analysis

Descriptive statistics for the LuLi-Q-Tots demonstrated variability, with prescribed scores ranging from 1 to 5 (Table 2). The minimum prescribed score was 1, and the maximum was 5 (Table 2). Mean scores ranged from 2.0 to 2.7, indicating general agreement with statements. The physical health domain, comprising 32 items, had a mean score of 2.4, while the emotional health domain (7 items) averaged 2.3. Behavioural expressions recorded the highest mean score at 2.7, whereas the feeling love domain had the lowest mean score of 2.0. Despite variations in the number of items across domains, standard deviations were consistent, indicating similar response variability. These findings suggest differences in participants’ experiences across domains, reflecting the multidimensional nature of HRQoL.

**Table 2.** Descriptive statistics of the HRQoL domain in Cohort 1.

	Cohort 1: 0–2 years (*n* = 50 participants)			
Domain	Mean	Standard deviation	Min	Max
Physical health (*n* = 32)	2.4	0.9	1	5
Emotional health	2.3	0.9	1	5
Behavioural expressions (*n* = 18)	2.7	1.0	1	5
Social well-being (*n* = 12)	2.2	0.8	1	5
Feeling love (*n* = 7)	2.0	0.7	1	5
Early development (*n* = 21)	2.1	0.7	1	5
Routine (*n* = 10)	2.4	0.9	1	5

HRQoL = health-related quality of life.

#### Item–total correlations

We conducted item–total correlations to evaluate the relationship between individual items and the overall scale score of the LuLi-Q-Tots measure. A total of 107 items were analysed. Forty-nine items across seven domains showed corrected item–correlations below 0.35, indicating weak correlations with the overall measure. Specifically, these included 9 (18.4%) items from the physical health domain, 2 (4.1%) from emotional health, 8 (16.4%) from behavioural expressions, 7 (14.3%) feeling love domain, 12 (24.5%) from early development, and 4 (8.2%) from the routine domain.

#### Internal consistency reliability

The LuLi-Q-Tots demonstrated excellent reliability, with a Cronbach’s alpha of (α = 0.93) across 107 items (Field, 2009). After removing 42 items, reliability improved to 0.95. Removing an entire domain, such as ‘Feeling love,’ based solely on poor correlations, was approached with caution. Reliability analysis showed acceptable reliability for 5 of the 7 subscales (α = 0.71–0.91). However, the ‘Social Well-being’ subscale (α = 0.659) and ‘Feeling love’ (α = 0.624) fell slightly below the acceptable threshold (Table 3).

**Table 3.** LuLi-Q-Tots scales statistics of the pilot sample (with the number of items) after removing selected items.

Domain/subscale	Items *n*	Mean score^*^	Internal consistency^†^
Physical health	24	2.3	0.905
Emotional health	5	2.3	0.832
Behavioural expressions	10	2.6	0.830
Feeling love	7	2.0	0.624
Social-well-being	5	2.3	0.659
Early development	9	2.1	0.820
Routine	5	2.5	0.706

* Possible range 1–5, with lower scores indicative of greater agreement.

† Cronbach’s alpha.

### Preliminary HRQoL using the LuLi-Q-Tots

Data were collected from baseline to long-term follow-ups. Overall, 15 (30%) of caregivers reported that their children had a limited appetite, 14 (28%) noted difficulty gaining weight, shorter stature, and a dislike of medication (including its taste), and 12 (24%) reported sleep difficulties. Additionally, 30 (60%) of children became more fidgety or restless when ill, while 36 (72%) became more attached to their caregivers. Hospital unfamiliarity contributed to 32 (64%) children becoming more anxious around strangers. Family stress due to illness was reported by 24 (48%), while 38 (80%) reported a strong support network.

### Psychometric properties of the LuLi-Q-Pres measure

#### Floor and ceiling effects for the LuLi-Q-Pres measure

The LuLi-Q-Pres item measure assessed 131 items across 7 HRQoL domains. Results showed that 80 (61%) displayed significant floor and ceiling effects (Addendum 1). A ceiling effect occurs when scores cluster near the highest possible value, limiting the ability to detect subtle differences or improvements.^26^

All seven domains displayed both floor (score ≥ 1) and ceiling effects (score ≥ 5). In the physical health domain, 18 (54.5%) showed ceiling effects. The emotional health domain showed 10 (83.3%) items with ceiling effects, while the psychological domain saw 5 (16.1%) exhibiting both ceiling and floor effects. Among the social well-being items, 12 (70.6%) showed floor effects. The ‘feeling loved’ domain had 6 (85.7%) items with floor effects. In the preschool readiness domain, 18 (85.7%) items exhibited floor effects, while the routine domain had no floor or ceiling effects.

#### Item analysis

The descriptive statistics for the LuLi-Q-Pres measure demonstrated response variability across the domains (Table 4). Overall mean scores ranged from 1.8 to 2.4, indicating a general trend of agreement with the statements. The physical health domain had the highest mean score of 2.4, followed closely by the emotional health domain at 2.3. In contrast, the ‘Feeling Love’ domain exhibited the lowest mean score at 1.8. These findings highlight variability in responses, with the greatest agreement observed in the physical and emotional domains and the least agreement in the ‘Feeling Love.’

**Table 4.** Descriptive statistics of the HRQoL domain in Cohort 2.

Domain	Cohort 2: 3–5 years (*n* = 110 participants)			
	Mean	Standard deviation	Min	Max
Physical health (*n* = 33)	2.4	1.0	1	5
Emotional (*n* = 12)	2.3	1.0	1	5
Psychological (*n* = 31)	2.4	1.0	1	5
Social well-being (*n* = 7)	2.0	0.9	1	5
Feeling love (*n* = 7)	1.8	0.6	1	5
Preschool readiness (*n* = 21)	2.2	1.0	1	5
Routine (*n* = 10)	2.2	0.9	1	5

HRQoL = health-related quality of life.

#### Item–total correlations

An item analysis of all 131 items in the LuLi-Q-Pres measure identified 29 items with corrected item–total correlations below 0.35, indicating weak alignment with the overall measure score. Of these, 12 (41.4%) items were from the physical health domain, 2 (6.9%) from the emotional health domain, 8 (27.6%) from the psychological health domain, 4 (13.8%) from the social domain, 1 (3.4%) from the preschool readiness domain, and 3 (10.3%) from the routine domain. These findings underscore areas where certain items, particularly within the physical and psychological health domains, did not align with the overall construct. Items with poor correlations were subsequently removed to improve the reliability and validity of the measures.

#### Internal consistency reliability

The LuLi-Q-Pres demonstrated excellent reliability, with a Cronbach’s alpha of 0.96, which further improved to 0.97 after removing 29 items. Reliability analysis across the seven subscales showed acceptable reliability, with Cronbach’s alpha values ranging from 0.78 to 0.87, except for the routine subscale, which had a lower alpha of 0.67. It is anticipated that larger sample sizes could improve these coefficients. While factor analysis was not feasible due to the small sample size, this limitation will be addressed in future research involving a larger sample.

Table 5 presents the mean scores for all seven domains, highlighting the highest and lowest agreement areas. Emotional health, psychological health, and preschool readiness domains received the highest scores, indicating strong caregiver agreement with these items. In contrast, the ‘Feeling Love’ domain had the lowest scores, suggesting that participants more frequently ‘disagreed’ with these statements. These results underscore variability in responses across domains, with notable strengths in emotional and psychological health and areas for potential improvement in the perceived ‘Feeling Love.’

**Table 5.** LuLi-Q-Pres scale statistics of the pilot sample (with the number of items) after removing selected items.

Domain/subscale	Items *n*	Mean score^*^	Internal consistency^†^
Physical health	19	2.0	.877
Emotional health	10	2.2	.782
Psychological	22	2.2	.896
Social well-being	14	2.0	.908
Feeling love	7	1.8	.885
Preschool readiness	20	2.2	.870
Routine	7	2.1	.667

* Possible range 1–5, with lower scores indicative of greater agreement.

† Cronbach’s alpha.

### Preliminary HRQoL among the LuLi-Q-Pres cohort

The older cohort demonstrated less pronounced physical health impacts than the younger group. A total of 24 (21.8%) participants expressed concerns about their children’s weight gain. Emotional strain was evident, with over 72 children (65%) experiencing separation difficulties from their caregivers and 58 (52.7%) displaying strong attachments to caregivers and extended family.

Behavioural changes were also common, with nearly 43 (40%) of children becoming more fidgety and over 40 (36%) becoming clingier during episodes of illness. Family dynamics were affected, as 46 (41%) of caregivers reported increased stress due to their child’s health issues. More than 102 (92%) observed jealousy in their children. Additionally, 25 (23%) struggled to keep up with their peers, suggesting potential developmental delays.

## DISCUSSION

This study presents a pilot evaluation of two measures developed to assess HRQoL in young children with respiratory illnesses—one designed for ages 0–2 years and another for ages 3–5 years. The analysis underscores the reliability and validity of the LuLi-Q measures while highlighting the critical need to evaluate floor and ceiling effects to ensure item appropriateness across different age groups.

In the 0–2-year cohort, minimal floor and ceiling effects were observed, suggesting that the LuLi-Q-Tots measure effectively captures HRQoL without significant bias. Conversely, the 3–5-year cohort exhibited substantial floor and ceiling effects, indicating issues with item sensitivity and the need for further refinement. Strategies to address these challenges include revising the response scale, incorporating more discriminative items, or adjusting question ranges better to capture the full spectrum of children’s experiences. These findings align with the literature emphasising the necessity for age-appropriate and context-sensitive measures to accurately assess HRQoL in young children, particularly in LMICs where socio-economic factors significantly influence perceived quality of life.^9,27^

Descriptive statistics and item–total correlations revealed variability in HRQoL experiences, with some items not aligning well with the overall construct. The internal consistency reliability of the seven domains in both the LuLi-Q-Tots and LuLi-Q Pres measures exceeded the recommended minimum alpha coefficient of 0.70.^23,24^ Removing poorly performing items further improved internal consistency, as indicated by higher Cronbach’s alpha scores.

The preliminary HRQoL findings illustrate the profound impact of symptomatic children with presumptive TB on young children’s HRQoL, extending beyond physical symptoms to encompass emotional, behavioural, and social challenges. The differences between the two cohorts highlight age-specific vulnerabilities, with younger children exhibiting attachment-related anxieties and older children facing more complex psychological and social issues. The high prevalence of daily medication use (54%) reported for children aged 0–2 years highlights the significant burden of treatment management, with potential implications for adherence, side effects, and disruptions to daily routines, all of which can adversely affect both the child’s and caregiver’s HRQoL. A study conducted among children with PTB highlighted the intricate interplay of factors such as caregiver-child separation, long-term hospitalisation, and social adversity among children with TB.^28^ While standard HRQoL measures capture some aspects of these children’s experiences, a more comprehensive approach is needed to fully understand and address the multidimensional effects of TB on young children and their families. Incorporating qualitative interviews will provide insights into nuanced experiences.

The study’s strengths include the development of two age-specific HRQoL measures based on two item banks with tested psychometric properties. The use of age-specific item banks ensures that the measures are developmentally appropriate, which is crucial for accurately capturing HRQoL in these age groups. However, several limitations should be acknowledged. While sufficient for preliminary analysis, the presence of floor and ceiling effects in the older cohort and the study’s sample size may not be large enough to generalise findings or conduct advanced statistical analyses such as factor analysis. Future research should focus on developing a scoring system for the LuLi-Q measures to show better or poorer HRQoL. This analysis would provide deeper insights into the underlying structure of the HRQoL measure and identify whether items group into coherent domains as expected, revealing patterns or latent constructs not captured by simple item–total correlations.

In conclusion, this study represents a foundational step toward creating sensitive and reliable HRQoL measures for young children with presumptive TB. The insights gained from this preliminary analysis will inform the ongoing refinement and validation of the LuLi-Q-Tots and LuLi-Q-Pres measures, ultimately contributing to improved patient-centred care and outcomes for this vulnerable population. Future research should focus on expanding sample sizes, refining item content, and validating the factor structure to enhance the robustness and applicability of these measures.

## References

[1] Ahmed R, The epidemiology of noncommunicable respiratory disease in sub-Saharan Africa, the Middle East, and North Africa. Malawi Med J. 2017;29(2):203–211. 28955434 10.4314/mmj.v29i2.24 PMC5610297

[2] Marangu D, Zar HJ. Childhood pneumonia in low-and-middle-income countries: an update. Paediatr Respir Rev. 2019;32:3–9. 31422032 10.1016/j.prrv.2019.06.001 PMC6990397

[3] Wang X, Global burden of respiratory infections associated with seasonal influenza in children under 5 years in 2018: a systematic review and modelling study. Lancet Glob Health. 2020;8(4):e497–e510. 32087815 10.1016/S2214-109X(19)30545-5 PMC7083228

[4] van der Zalm MM, High burden of viral respiratory co-infections in a cohort of children with suspected pulmonary tuberculosis. BMC Infect Dis. 2020;20(1):1–10. 10.1186/s12879-020-05653-9 PMC7716283 33276721

[5] Marais BJ, The burden of childhood tuberculosis and the accuracy of community-based surveillance data. Int J Tuberc Lung Dis. 2006;10(3):259–263. 16562704

[6] Hordijk J, Health-related quality of life of children and their parents 6 months after children’s critical illness. Qual Life Res. 2020;29(1):179–189. 31691884 10.1007/s11136-019-02347-x PMC6962289

[7] Nkereuwem E, ‘Yes! We can end TB,’ but remember the sequelae in children. Lancet Respir Med. 2024;12(May):348–350. 38527484 10.1016/S2213-2600(24)00078-X

[8] van der Zalm MM, Impaired lung function in adolescents with pulmonary tuberculosis during treatment and following treatment completion. eClinicalMedicine. 2024;67:102406. 38261903 10.1016/j.eclinm.2023.102406 PMC10796966

[9] Anthony MG, Caregivers’ perspectives on health-related quality of life in young children with TB and respiratory illnesses. Public Health Action. 2022;12(1):201–205. 36561904 10.5588/pha.22.0038 PMC9716816

[10] Cesnales NI, Health-related quality of life among people living with HIV/AIDS receiving case management. J HIV AIDS Soc Serv. 2016;15(2):202–215.

[11] Germain N, Measuring health-related quality of life in young children: how far have we come? J Mark Access Health Policy. 2019;7(1):1618661. 10.1080/20016689.2019.1618661 PMC6534256 31156762

[12] Verstraete J, How does the EQ-5D-Y Proxy version 1 perform in 3, 4 and 5-year-old children? Health Qual Life Outcomes. 2020;18(1):1–10. 32448278 10.1186/s12955-020-01410-3 PMC7247261

[13] Dewandel I, UMOYA: a prospective longitudinal cohort study to evaluate novel diagnostic tools and to assess long-term impact on lung health in South African children with presumptive pulmonary TB—a study protocol. BMC Pulm Med. 2023;23(1):1–11. 36949477 10.1186/s12890-023-02329-3 PMC10032249

[14] Achoki T, Health trends, inequalities and opportunities in South Africa’s provinces, 1990–2019: findings from the Global Burden of Disease 2019 Study. J Epidemiol Community Health. 2022;76(5):471–481. 35046100 10.1136/jech-2021-217480 PMC8995905

[15] van Niekerk M, Clinical paediatric tuberculosis research: field experiences from Cape Town, South Africa. 2023;(December):5–7.

[16] Allwood BW, Clinical evolution, management and outcomes of patients with COVID-19 admitted at Tygerberg Hospital, Cape Town, South Africa: a research protocol. BMJ Open. 2020;10(8):1–6. 10.1136/bmjopen-2020-039455 PMC7462165 32868368

[17] Weimann A, Oni T. A systematised review of the health impact of urban informal settlements and implications for upgrading interventions in South Africa, a rapidly urbanising middle-income country. Int J Environ Res Public Health. 2019;16(19):1–17. 10.3390/ijerph16193608 PMC6801583 31561522

[18] Masekela R, Vanker A. Lung health in children in sub-Saharan Africa: addressing the need for cleaner air. Int J Environ Res Public Health. 2020;17(17):1–13. 10.3390/ijerph17176178 PMC7504680 32858786

[19] Shi T, Global, regional, and national disease burden estimates of acute lower respiratory infections due to respiratory syncytial virus in young children in 2015: a systematic review and modelling study. Lancet. 2017;390(10098):946–958. 28689664 10.1016/S0140-6736(17)30938-8 PMC5592248

[20] Harris PA, Research electronic data capture (REDCap)—a metadata-driven methodology and workflow process for providing translational research informatics support. J Biomed Inform. 2009;42(2):377–381. 18929686 10.1016/j.jbi.2008.08.010 PMC2700030

[21] Harris PA, The REDCap consortium: building an international community of software platform partners. J Biomed Inform. 2019;95:103208. 31078660 10.1016/j.jbi.2019.103208 PMC7254481

[22] McHorney CA, Tarlov AR. Individual-patient monitoring in clinical practice: are available health status surveys adequate? Qual Life Res. 1995;4(4):293–307. 7550178 10.1007/BF01593882

[23] Field A. Discovering statistics using SPSS. 3^rd^ ed. London, UK: Sage Publications, 2009.

[24] Field A. Discovering statistics using SPSS. 4^th^ ed. London, UK: Sage Publications, 2013.

[25] DeVellis RF. Scale development: theory and applications. 4^th^ ed. Thousand Oaks, CA, USA: Sage Publications, 2017.

[26] Terwee CB, Quality criteria were proposed for measurement properties of health status questionnaires. J Clin Epidemiol. 2007;60(1):34–42. 17161752 10.1016/j.jclinepi.2006.03.012

[27] Murugappan MN, Floor and ceiling effects in the EORTC QLQ-C30 physical functioning subscale among patients with advanced or metastatic breast cancer. Cancer. 2022;128(4):808–818. 34634139 10.1002/cncr.33959 PMC9923627

[28] Meyerson K. Exploring caregivers and health workers’ perceptions on the effects of caregiver-child separation during long-term hospitalisation for MDR-TB in the Western Cape: a qualitative study. Tygerberg, South Africa: Stellenbosch University, 2019.

